# Micronutrient malnutrition and wasting in adults with pulmonary tuberculosis with and without HIV co-infection in Malawi

**DOI:** 10.1186/1471-2334-4-61

**Published:** 2004-12-21

**Authors:** Monique van Lettow, Anthony D Harries, Johnny J Kumwenda, Ed E Zijlstra, Tamara D Clark, Taha E Taha, Richard D Semba

**Affiliations:** 1 Johns Hopkins University School of Medicine, Baltimore, USA; 2 National Tuberculosis Control Programme, Lilongwe, Malawi; 3 College of Medicine, Blantyre, Malawi; 4 Johns Hopkins University School of Public Health, Baltimore, USA

## Abstract

**Background:**

Wasting and micronutrient malnutrition have not been well characterized in adults with pulmonary tuberculosis. We hypothesized that micronutrient malnutrition is associated with wasting and higher plasma human immunodeficiency virus (HIV) load in adults with pulmonary tuberculosis.

**Methods:**

In a cross-sectional study involving 579 HIV-positive and 222 HIV-negative adults with pulmonary tuberculosis in Zomba, Malawi, anthropometry, plasma HIV load and plasma micronutrient concentrations (retinol, α-tocopherol, carotenoids, zinc, and selenium) were measured. The risk of micronutrient deficiencies was examined at different severity levels of wasting.

**Results:**

Body mass index (BMI), plasma retinol, carotenoid and selenium concentrations significantly decreased by increasing tertile of plasma HIV load. There were no significant differences in plasma micronutrient concentrations between HIV-negative individuals and HIV-positive individuals who were in the lowest tertile of plasma HIV load. Plasma vitamin A concentrations <0.70 μmol/L occurred in 61%, and zinc and selenium deficiency occurred in 85% and 87% respectively. Wasting, defined as BMI<18.5 was present in 59% of study participants and was independently associated with a higher risk of low carotenoids, and vitamin A and selenium deficiency. Severe wasting, defined as BMI<16.0 showed the strongest associations with deficiencies in vitamin A, selenium and plasma carotenoids.

**Conclusions:**

These data demonstrate that wasting and higher HIV load in pulmonary tuberculosis are associated with micronutrient malnutrition.

## Background

Approximately one-third of the world's population is infected with *Mycobacterium tuberculosis*, and the majority live in less developed countries where human immunodeficiency virus (HIV) infection is spreading rapidly. The World Health Organization (WHO) estimates that the number of new cases of tuberculosis and the proportion with coexisting HIV infection will continue to increase [1]. Immunosuppression increases the risk of developing clinical tuberculosis, which contributes to the increased prevalence of tuberculosis in association with HIV infection. Malnutrition and wasting are associated with tuberculosis, and co-infection with HIV and tuberculosis may potentially exacerbate the wasting that occurs in tuberculosis or HIV infection alone [2-5]. Micronutrient deficiencies have been described in individuals with tuberculosis [6-17] and in those with HIV infection [17-23]. Several cross-sectional studies suggest that patients with tuberculosis are at high risk of deficiencies of vitamin A [7,10-12], thiamin [13], vitamin B_6 _[14], folate [6,15], vitamin E [16], and zinc [10]. Poor selenium status has recently been shown to increase the risk of developing mycobacterial disease among HIV-infected injection drug users [24], but selenium status among HIV-infected adults with pulmonary tuberculosis has not been well characterized. Selenium plays an important role in the selenoenzyme glutathione peroxidase that protects cells against free radical damage and oxidative stress.

The relationship between severity of HIV disease and micronutrient malnutrition needs further elucidation. Such information would help identify subgroups that might benefit the most from nutritional interventions. Plasma HIV load was used as an indicator of severity of HIV disease, as HIV load tends to be higher in more active HIV disease. We hypothesized that wasting in pulmonary tuberculosis is associated with micronutrient malnutrition and that HIV-infected adults with pulmonary tuberculosis who have more active HIV disease, as reflected by higher HIV load, also have more severe micronutrient malnutrition. To test these hypotheses, we conducted a cross-sectional study to examine the relationship between wasting and micronutrient malnutrition in HIV-positive and HIV-negative adults with pulmonary tuberculosis in Zomba, Malawi.

## Methods

The study population consisted of adults who presented with new sputum-positive pulmonary tuberculosis in Zomba Central Hospital between July 1999 and April 2003. Subjects were offered HIV testing and were screened for HIV antibodies after signing a written informed consent form. All subjects were given appropriate pre- and post-test HIV counseling. Subjects commenced treatment after enrollment and received standard short course chemotherapy for tuberculosis as per guidelines of the Malawi National Tuberculosis Program [25]. Adults with a previous history of treated pulmonary tuberculosis were excluded. Three sputum samples from each subject were examined with Auramine-O dark-fluorescent staining method. Sputum positive pulmonary tuberculosis was considered proven when at least one out of three sputum stains showed acid-fast bacilli. HIV infection was diagnosed on the basis of a positive rapid test (Determine 1/2 Rapid test by Abbott, Abbott Laboratories, Johannesburg, SA) and confirmed by a positive enzyme-linked immunosorbent assay for HIV-1 antibodies (Wellcozyme; Wellcome Diagnostics, Dartford, Kent, UK). Plasma HIV load was measured using quantitative HIV-1 RNA PCR (Roche Amplicor Monitor, version 1.5, Branchburg, NJ, USA) with a sensitivity limit of 400 HIV RNA copies mL. CD4^+ ^lymphocyte counts were not conducted due to limited resources. None of the participants were taking antiretroviral treatment.

The protocol was approved by the institutional review boards at the Johns Hopkins School of Medicine (Baltimore, Maryland, USA) and the College of Medicine, University of Malawi (Blantyre, Malawi), with final approval by the Office for Protection from Research Risk of the National Institutes of Health.

### Nutritional assessment

Body weight was determined to the nearest 0.1 kg using an adult balance (Seca 700 balance, Seca Corporation, Hanover, MD, USA), and standing height was determined to the nearest cm. Body mass index (BMI) was calculated as body weight/height^2^.

### Plasma micronutrient concentrations

A venous blood sample was collected by venipuncture (Sarstedt Monovette, Newton, NC). Blood samples were shielded from bright light and immediately aliquoted and stored in cryotubes at -70°C. α-carotene, β-carotene, β-cryptoxanthin, lycopene, lutein, zeaxanthin, retinol, and α-tocopherol concentrations were measured in 100 uL of plasma by high performance liquid chromatography using a modified method from the Nutrition Laboratory, Inorganic Toxicology and Nutrition Branch Division of Laboratory Sciences, National Center of Environmental Health, Centers of Disease Control and Prevention (Rosemary Schleicher, personal communication) [27]. Total plasma carotenoids were defined as the sum of α-carotene, β-carotene, β-cryptoxanthin, lycopene, lutein and zeaxanthin in μmol/L. Plasma trace element concentrations were measured using a Perkin Elmer model AAnalyst 600 atomic absorption spectrometer equipped with Zeeman background correction, a THGA graphite furnace, and an AS800 auto sampler (Perkin Elmer Corp., Norwalk, CT). Quality control was assessed by repeated analysis of pooled human plasma controls run at the beginning and the end of each analysis. Standard curves were run periodically using standard reference material 986C (National Institute of Standards and Technology, Gaithersburg, MD). Throughout all analyses, the plasma samples were run in a masked fashion.

### Data and statistical analysis

Data and statistical analysis were conducted using SAS 8.01 (SAS Institute Cary, NC, USA) and SPSS 9.0 (SPSS, Inc., Chicago, IL, USA). Comparisons between groups were made using *t*-tests and nonparametric Mann-Whitney *U*-tests. Univariate analysis of variance was used to test for linear trends across categories of plasma HIV load and BMI.

HIV load was categorized into tertiles. HIV negative subjects were assigned a fourth category of HIV load (category 0) when groups were merged for analysis. Nutritional status was assessed in adults with pulmonary tuberculosis with and without HIV co-infection. Subjects were separated into groups according to their extent of wasting. Mild wasting was defined as BMI 17.0–18.49, moderate wasting as BMI 16.0–16.99, and severe wasting as BMI <16.0, conform the WHO strata for BMI grading of severity of malnutrition [27].

Plasma retinol <0.70 μmol/L was considered consistent with vitamin A deficiency [28]. Vitamin E deficiency was defined as plasma α-tocopherol <11.6 μmol/L [28]. Zinc deficiency was defined as plasma zinc <11.5 μmol/L and selenium deficiency as plasma selenium <0.89 μmol/L [28]. Because there is no standard cut-off for deficiency of carotenoids, we divided total plasma carotenoids into quartiles, with the lowest quartile considered to be the most consistent with deficiency.

To examine the risk of micronutrient deficiencies at different severity level of wasting, logistic regression models were fitted with retinol <0.70, α-tocopherol <11.6, zinc <11.5, selenium <0.89, and the lowest quartile of total carotenoids as the outcome variable. Multivariate logistic regression models were conducted to adjust for sex, age and HIV load. A significance level of *P *< 0.01 was used in this study.

## Results

The study population consisted of 579 HIV-positive and 222 HIV-negative adults with sputum-positive pulmonary tuberculosis. Among the total study population, 66% (232/352) of male and 77% (347/449) of female participants were HIV-positive. The mean age among all subjects was 33 years (range 18–59 years). The majority of subjects were wasted, as 59% of subjects had a BMI <18.5, 32% of subjects had a BMI <17.0, and 17% of all subjects were severely wasted as defined by BMI<16.0. Plasma retinol concentrations <0.70 μmol/L occurred in 61% of all subjects. Vitamin E, zinc, and selenium deficiency occurred in 13%, 85% and 87% respectively. Table 1 shows characteristics of study participants, such as sex, age, BMI, and plasma carotenoids, retinol, α-tocopherol, zinc and selenium by categories of plasma HIV load. BMI, plasma retinol, total carotenoids and selenium concentrations decreased by increasing plasma HIV load. Age, the proportion of individuals with BMI <18.5, BMI <16.0 and selenium deficiency were increased with increasing plasma HIV load. Plasma α-tocopherol, zinc or the proportion of individuals with vitamin A, vitamin E, or zinc deficiencies were not significantly different across the categories of plasma HIV load. When exploring across the categories separately, there were no significant differences between HIV-negative individuals compared with HIV-positive individuals in the lowest tertile of viral load.

Table 2 shows adjusted odds ratios (O.R.) and 95% confidence intervals (C.I.) for independent associations between wasting and micronutrient deficiencies. Wasting defined as BMI<18.5 was associated with vitamin A deficiency, low plasma carotenoids and selenium deficiency. The odds ratio for an independent association with vitamin A deficiency was 2.86 (95% C.I. 2.11–3.89) when adjusted for sex, age, and plasma HIV load. The adjusted odds ratio for an independent association with the lowest quartile of total carotenoids was 2.96 (95% C.I. 1.99–4.44). The adjusted odds ratio for an independent association with selenium deficiency was 1.59 (95% C.I. 1.04–2.43). When separating severity levels of wasting; mild wasting did not show association with deficiencies, moderate wasting was associated with vitamin A deficiency and severe wasting was significantly associated with vitamin A deficiency, low plasma carotenoids and selenium deficiency. (Table 2) Wasting was not associated with vitamin E or zinc deficiency.

**Figure 1:**
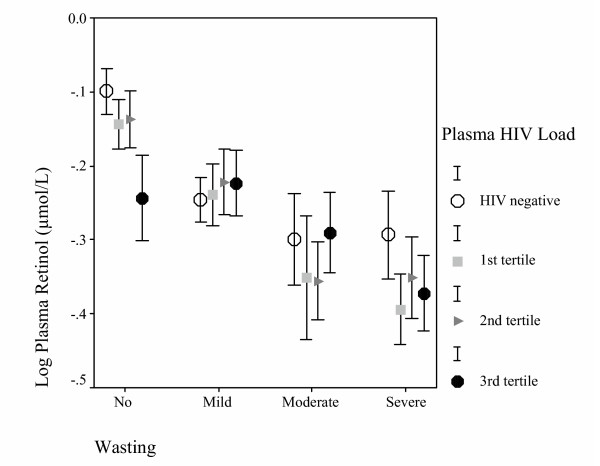
Log-transformed mean plasma retinol concentrations with 95% C.I. are depicted by severity of wasting and plasma HIV load. Among the not-wasted adults with pulmonary tuberculosis log mean plasma retinol concentration significantly decrease with the increase of plasma HIV load (*P *= 0.004). Among those with mild, moderate and severe wasting this linear trend did not reach significance. Among the HIV negative adults with pulmonary tuberculosis, log mean plasma retinol concentration significantly decrease with the increasing severity of wasting (*P *= 0.0001). The same trend appears among those in the 1^st^, 2^nd ^and 3^rd ^tertile of HIV load; *P *= 0.0001, *P *= 0.0001 and *P *= 0.01 respectively.

**Figure 2:**
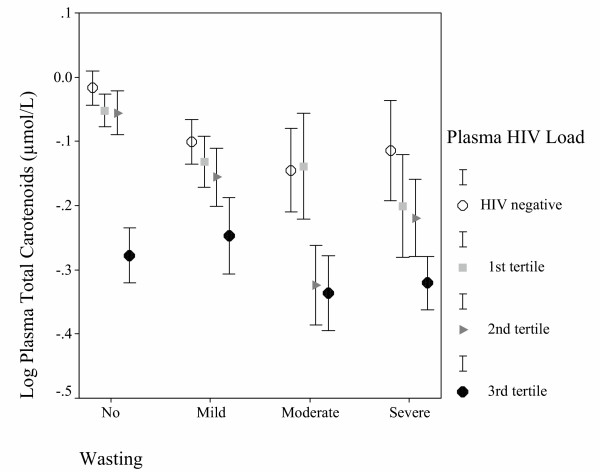
Log-transformed mean plasma total carotenoid concentrations with 95% C.I. are depicted by severity of wasting and plasma HIV load. Among not-wasted, mildly wasted, moderately wasted and severely wasted log mean plasma total carotenoid concentrations significantly decrease with the increase of plasma HIV load (*P *= 0.0001, *P *= 0.002, *P *= 0.001 and *P *= 0.001, respectively). Among the HIV negative adults, and those in the 1^st ^and 2^nd ^tertile of plasma HIV load, log mean plasma total carotenoid concentrations significantly decreased with the increasing severity of wasting (*P *= 0.007, *P *= 0.002 and *P *= 0.0001, respectively). This trend did not reach significance among those in the 3^rd ^tertile of plasma HIV load.

**Figure 3:**
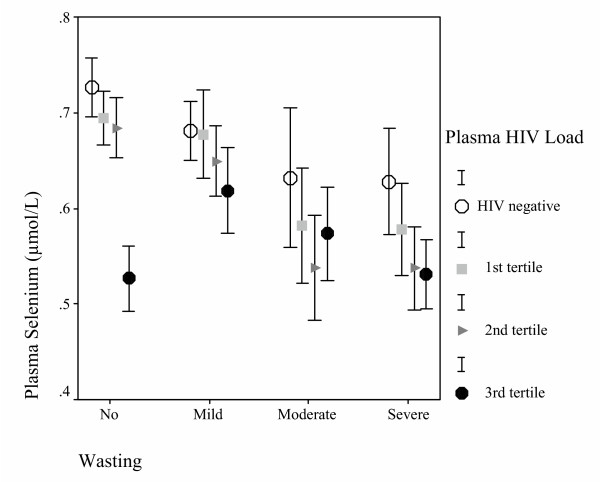
Mean plasma selenium concentrations with 95% C.I. are depicted by severity of wasting and plasma HIV load. Among not-wasted and severely wasted adults, mean plasma selenium concentrations significantly decrease with the increase of plasma HIV load (*P *= 0.0001 and *P *= 0.03, respectively). This trend did not reach significance among those with mild and moderate wasting. Among the HIV negative adults, and those in the 1^st ^and 2^nd ^tertile of plasma HIV load, mean plasma selenium concentrations significantly decreased with the increasing severity of wasting (*P *= 0.02, *P *= 0.008 and *P *= 0.0001, respectively). This trend did not reach significance among those in the 3^rd ^tertile of plasma HIV load.

Figures 1, 2 and 3 show plasma retinol, total plasma carotenoids, and plasma selenium concentrations with 95% C.I by severity of wasting and categories of plasma HIV load.

Plasma retinol concentrations significantly decreased with the increase of plasma HIV load among non-wasted adults with pulmonary tuberculosis (*P *= 0.004). Total carotenoid concentrations significantly decreased with the increase of plasma HIV load among non-wasted, mildly wasted, moderately wasted and severely wasted adults (*P *= 0.0001, *P *= 0.002, *P *= 0.001 and *P *= 0.001, respectively). Selenium concentrations decreased significantly with the increase of plasma HIV load among non-wasted and severely wasted adults with pulmonary tuberculosis (*P *= 0.0001 and *P *= 0.03, respectively). Among the HIV negative adults and those in the 1^st ^and 2^nd ^tertile of HIV load, plasma retinol, total carotenoids and selenium concentrations significantly decrease with the increasing severity of wasting. Among those in the 3^rd ^tertile of HIV load, only plasma retinol concentrations significantly decreased with the increasing severity of wasting. This trend did not reach significance for plasma carotenoid and selenium concentrations.

## Discussion

The present study shows that micronutrient malnutrition and wasting are more severe in adults with pulmonary tuberculosis who have higher plasma HIV load. The association between high plasma HIV load and nutrient deficiencies was strongest for the major plasma carotenoids and selenium. Overall in this study population, both HIV-positive and HIV-negative adults with pulmonary tuberculosis were extremely malnourished as indicated by BMI and plasma micronutrient concentrations. About one-third of the adults in this study had a BMI <17.0, a cut-off that is predictive of mortality in adults co-infected with tuberculosis and HIV [29].

To our knowledge, this is the first study to demonstrate that selenium status is extremely poor among HIV-infected adults with pulmonary tuberculosis, and that the extent of selenium deficiency is associated with higher plasma HIV load. This observation may be of potential importance because selenium deficiency has been associated with increased mortality during HIV infection [30], and selenium supplementation for HIV-infected adults has been shown to reduce morbidity [31]. In the present study, selenium deficiency occurred in 87% of the participants, which, to our knowledge, may be the highest prevalence of selenium deficiency reported in an HIV-infected group of patients. It is unknown whether selenium supplementation will reduce morbidity and mortality among HIV-infected adults with pulmonary tuberculosis.

Carotenoids are among the most important dietary antioxidants found in human plasma, and this study shows that poor carotenoid status was associated with higher HIV load and with wasting. Plasma carotenoid concentrations are widely considered to be the most accurate indicator of dietary carotenoid intake [32]. It is not known whether adults with pulmonary tuberculosis and higher HIV load have lower plasma carotenoid concentrations because of increased oxidative stress, or whether these individuals are sicker and unable to consume enough carotenoid-rich foods. Further studies are needed in the future to address dietary intake of carotenoids in HIV-infected adults with pulmonary tuberculosis.

Low BMI is a known risk factor for mortality [5,29], and the present study showed that the risk of micronutrient deficiencies is highest in those with low BMI.

HIV-infected adults with wasting and high viral load were at the highest risk of more severe micronutrient malnutrition, suggesting that this subgroup might potentially benefit the greatest from nutritional interventions.

The cross sectional design of this study restricts our conclusions and does not provide information on whether poor nutritional status is a predictor of more severe pulmonary tuberculosis. It is unknown whether nutritional interventions will slow progression of disease or reduce wasting associated with morbidity and mortality if added to tuberculosis chemotherapy. Controlled clinical trials currently in progress in developing countries should help provide insight into the role of micronutrient supplementation for HIV-positive and HIV-negative adults with pulmonary tuberculosis.

## Conclusions

The present study shows that micronutrient malnutrition and wasting are more severe in adults with pulmonary tuberculosis who have higher HIV load. The association between high plasma HIV load and nutrient deficiencies was strongest for the major plasma carotenoids and selenium. Further longitudinal investigations are needed to determine whether deficiencies in micronutrients are independent risk factors for increased morbidity and mortality.

## Competing interests

The author(s) declare that they have no competing interests.

## Authors' contributions

Overall guidance and initial study design was provided by RS. MvL has been in charge of the collection and analysis of data and writing of the manuscript. Provision of advice was given by AH, JK, EZ, TC and TT. All authors read and approved the final manuscript.

## Pre-publication history

The pre-publication history for this paper can be accessed here:



## Figures and Tables

**Table 1:** Characteristics of adults presenting with pulmonary tuberculosis in Zomba, Malawi – by plasma HIV load

**Characteristic^1^**	**HIV negative**	HIV positive* Plasma HIV Load (copies/mL)			*P*-value**
		
	0	≤ 133 200	133 200 – 406 000	> 406 000	
	**n = 222**	**n = 185**	**n = 186**	**n = 186**	
Sex (% Female)	45.9	63.2	58.1	55.9	0.07
Age (years)	33 ± 12	32 ± 12	33 ± 12	33 ± 12	0.01
Body mass index (BMI) (wt/ht^2^)	18.6 ± 2.9	19.0 ± 2.6	18.3 ± 3.0	17.3 ± 2.7	0.0001
Wasting:^2 ^No, BMI ≥ 18.5	42.3	51.4	41.9	30.1	0.005
Mild, BMI 17.0–18.49 (%)	32.0	27.0	22.6	24.2	0.04
Moderate, BMI 16.0–16.99 (%)	13.1	10.3	17.2	18.8	0.04
Severe, BMI <16.0 (%)	12.6	11.4	18.3	26.9	0.001
Retinol (μmol/L)	0.636 (0.367, 1.104)	0.603 (0.336, 1.085)	0.585 (0.321, 1.066)	0.522 (0.157, 1.738)	0.001
Vitamin A deficiency, retinol <0.70 μmol/L (%)^3^	58.6	57.3	64.0	66.7	0.05
Total Carotenoids (μmol/L)^4^	0.846 (0.490, 1.459)	0.795 (0.476, 1.329)	0.700 (0.385, 1.279)	0.509 (0.279, 0.929)	0.0001
α-tocopherol (μmol/L)	15.18 (11.71, 19.65)	14.90 (11.60, 19.16)	15.66 (11.85, 20.71)	16.07 (11.53, 22.40)	0.02
Vitamin E def., α-tocopherol <11.6 μmol/L (%)^3^	13.1	14.6	11.8	12.4	0.67
Zinc (μmol/L)	8.95 (7.01, 11.43)	8.83 (6.94, 11.25)	8.49 (6.44, 11.19)	9.15 (6.47, 12.94)	0.82
Zinc deficiency, zinc <11.5 μmol/L (%)^3^	84.2	88.1	87.6	77.4	0.09
Selenium (μmol/L)	0.687 ± 0.23	0.664 ± 0.22	0.624 ± 0.22	0.559 ± 0.21	0.0001
Selenium deficiency, selenium<0.89 μmol/L(%)^3^	79.7	84.9	90.3	92.5	0.001

^1 ^Mean ± SD for continues variables with normal distribution, geometric mean (lower, upper SD) when distribution was not normal

^2 ^Grading based on WHO Expert report, reference 27.

^3 ^Cut-offs based on reference 28.

^4^α-carotene + β-carotene + β-cryptoxanthin + lycopene + lutein + zeaxanthin

* HIV load could not be determined for 21 individuals.

* *ANOVA, linear trend across the 4 categories of plasma HIV load.

**Table 2:** Risk of micronutrient deficiencies at different severity levels of wasting in adults with pulmonary tuberculosis with and without HIV co-infection.

	**Mild wasting**		**Moderate wasting**		**Severe wasting**	
	BMI 17.0–18.49		BMI 16.0–16.99		BMI <16.0	
**Deficiency**	O.R. (95% C.I.)*	***P*-value**	O.R. (95% C.I.)*	***P*-value**	O.R. (95% C.I.)*	***P*-value**
Vitamin A deficiency	0.81 (0.58–1.13)	0.20	1.59 (1.03–2.47)	0.03	3.51 (2.19–5.72)	0.0001
Lowest quartile of Total Carotenoids	0.92 (0.62–1.36)	0.67	2.46 (1.57–3.85)	0.0001	1.82 (1.18–2.83)	0.007
Vitamin E deficiency	0.86 (0.54–1.37)	0.54	1.24 (0.70–2.18)	0.46	1.13 (0.64–1.97)	0.68
Zinc deficiency	0.71 (0.45–1.15)	0.17	0.61 (0.37–1.00)	0.05	0.76 (0.46–1.24)	0.27
Selenium deficiency	1.59 (1.04–2.43)	0.03	1.16 (0.62–2.17)	0.65	3.25 (1.38–7.62)	0.007

* Adjusted for sex (male), age (per year) and HIV load (quartiles, where category 0 is HIV negative and category 3 is the highest.
